# Multicolor Hair Dyeing with Biocompatible Dark Polyphenol Complex-Integrated Shampoo with Reactive Oxygen Species Scavenging Activity

**DOI:** 10.3390/biomimetics8060469

**Published:** 2023-10-01

**Authors:** Tae Min Kim, Hyun Jeong Won, Jun-Ho Yang, Hayeon Jo, A Hyeon Kim, Dohyun Nam, Seul Gi Kim, Eun-Jung Jin, Heung Jin Bae, Sung Young Park

**Affiliations:** 1 Department of Chemical and Biological Engineering, Korea National University of Transportation, Chungju 27469, Republic of Korea; ktm1120@a.ut.ac.kr (T.M.K.); dnjsswh@ut.ac.kr (H.J.W.); yeon81037@ut.ac.kr (H.J.); k.seulgi9797@gmail.com (S.G.K.); 2 Department of Biological Sciences, College of Health Sciences, Wonkwang University, Iksan 54538, Republic of Korea; skyjun96@gmail.com (J.-H.Y.); dhnam0120@gmail.com (D.N.); 3 Department of IT and Energy Convergence (BK21 FOUR), Korea National University of Transportation, Chungju 27469, Republic of Korea; dkgusaks@naver.com; 4 MODAMODA Corporation, Ltd., Songpa-gu, Seoul 05546, Republic of Korea

**Keywords:** polyphenol, hair coating, ROS scavenging, multicolor shampoo

## Abstract

Hair dyeing has become a prevalent lifestyle trend, especially within the fashion industry. However, it possesses disadvantages, such as containing carcinogenic and toxic materials. In this study, we developed a biocompatible hair-dyeing technology using a shampoo with a dark polyphenol complex (DPC), referred to as S-DPC. The DPC was formed from a mixture of gallic acid and [1,1′-biphenyl]-2,2′,4,4′,5,5′-hexol and used to enhance both the stability of the hair coating and its ability to scavenge reactive oxygen species (ROS). Colloidal DPC particles play a pivotal role in the coating process of various hair dyes, ensuring the uniform coloring of human hair through intermolecular interactions such as hydrogen bonding. Owing to the effect of a polyphenol complex on hair coating, we observed improved antistatic performance and enhanced mechanical strength, resulting in a substantial increase in elongation at the breaking point from 33.74% to 48.85%. The multicolor S-DPC exhibited antioxidant properties, as indicated by its ROS-scavenging ability, including 2,2-diphenyl-1-picrylhydrazyl inhibition (87–89%), superoxide radical scavenging (84–87%), and hydroxyl radical scavenging (95–98%). Moreover, the in vitro analysis of the DPC revealed nearly 100% cell viability in live and dead assays, highlighting the remarkable biocompatibility of the DPC. Therefore, considering its effectiveness and safety, this biomaterial has considerable potential for applications in hair dyeing.

## 1. Introduction

Hair-coloring technology was developed by Hofmann in 1863 through the investigation of the browning phenomenon of *p*-phenylenediamine on various substrates exposed to oxidizing agents [1]. Since then, hair-coloring technology has been utilized primarily for cosmetic purposes and to change one’s natural hair color according to personal preferences. However, despite its popularity in the past decade, hair dyeing presents several concerns due to its potential toxicity and the presence of carcinogenic compounds. Notably, contact with the skin or inhalation may induce allergic responses [2,3]. Furthermore, most dyeing techniques involve a reduction–oxidation process, wherein hydrogen peroxide (H_2_O_2_) is used to cover the hair, particularly in coating processes [4]. However, the continuous application of H_2_O_2_ may harm the hair structure, resulting in reduced protection of the hair and making it susceptible to breaking. In the long term, H_2_O_2_ can suppress the hair follicle growth rate and induce scalp dermatitis [5,6,7], as H_2_O_2_ is a reactive oxygen species (ROS), similar to superoxide anion (O_2_^–^), hydroxyl radical (•OH), and singlet oxygen (^1^O_2_) [8,9,10]. Additionally, dye residues on the hair can be challenging to remove after their initial application, thereby limiting the reusability of hair dyes. Accordingly, hair dyes with desirable biocompatibility, ROS-scavenging properties, and the ability to color the hair uniformly hold promise for use in hair coloring.

Polyphenols are present in various sources, including fruits, vegetables, cereals, and beverages, and are defined as compounds possessing one or more hydroxyl (OH) groups linked to the main aromatic ring structure [11]. The functionalization of this structure can substantially influence the biological activity of polyphenols. Polyphenols play several vital roles in the body, including protection against UV radiation and pathogenic threats [12,13]. Furthermore, they exhibit diverse biological properties, including anti-inflammatory, bacteriostatic, and anticancer effects, which are associated with reduced risk of developing chronic illnesses, such as cardiovascular diseases and cancer [14,15,16]. Chemically, the hydroxyl groups of polyphenols neutralize ROS by donating electrons to the free radicals, thereby restricting the oxidative damage to biological compounds such as lipids, proteins, and DNA [17,18]. Additionally, polyphenols can be utilized for hair treatment, particularly in the treatment of alopecia, and for the protection of the hair from photobleaching caused by UVA, UVB, and visible irradiation [14,19]. Polyphenols may also moderate the amount of ROS on the hair. Considering the compatibility between polyphenols and the hair, it is reasonable to assume that polyphenols can be used for biocompatible hair-dyeing applications.

In this study, we invented a biocompatible dark polyphenol complex (DPC)-integrated shampoo (S-DPC) for moderating the ROS level of the hair and for homogenous hair dyeing. The DPC was prepared through the combination of gallic acid (GA), a compound that is present in tea leaves, and [1,1′-biphenyl]-2,2′,4,4′,5,5′-hexol (HDP), a type of cellulosic biomass, which was incorporated into a mixture of shampoo and various dyes for hair coloring and coating application [20,21]. Owing to the hydrophobicity of HDP, the addition of polyvinyl alcohol (PVA) and GA was crucial to enhancing hydrophilicity and dispersibility. Moreover, the DPC served as a coat-on approach to hair dyeing, which produced uniform hair color through molecular binding between the dyes and the hair surface. The inclusion of GA in the formula enhanced the antistatic properties while improving the mechanical strength and flexibility of the hair. Because polyphenols, such as GA, possess antioxidant qualities, it was reasonable to hypothesize that the DPC reduces ROS levels in the hair by scavenging them. Furthermore, an in vitro study of the DPC demonstrated exceptional biocompatibility, with approximately 99% of cells remaining viable in live and dead assays. This result indicates that our integrated biomaterial–shampoo hybrid holds potential for safe and effective hair dyeing.

## 2. Materials and Methods

### 2.1. Materials and Characterization

The base shampoo (MODAMODA Zero Gray Shampoo) was purchased from MODAMODA Corporation. GA, allura red AC (AR), brilliant blue FCF (Blue 1), naphthol blue black (NBB), rhodamine B (Rho-B), thiazolyl blue tetrazolium bromide (TBTB), PVA, 2,2-diphenyl-1-picrylhydrazyl (DPPH), salicylic acid, ferrous sulfate (FeSO_4_), H_2_O_2_, phosphate-buffered saline (PBS), trizma hydrochloride (tris-HCl), pyrogallol, and sodium hydroxide (NaOH) were procured from Sigma-Aldrich. HDP and caramel dyes (CC) were purchased from FNB Tech.

UV-visible (UV-Vis) spectra were obtained using an Optizen 2020UV spectrometer (Mecasys, Daejeon, South Korea). Fluorescence spectra were recorded using a fluorescence spectrometer (L550B; Perkin Elmer, Waltham, MA, USA). Fourier Transform infrared spectroscopy (FTIR) was performed using an FTIR spectrometer (Thermo Nicolet iS10) in the scan range of 600–4000 cm^−1^. Thermal gravimetric analysis (TGA) and differential scanning calorimetry (DSC) were performed using a DSC2910 differential thermal analyzer (TA Instruments, New Castle, DE, USA) at a heating rate of 10 °C/min under nitrogen (N_2_) atmosphere. Atomic force microscopy (AFM) was performed using an XE-100 atomic force microscope (PSIA, Sungnam, South Korea). Color specifications were observed using a Color Reader CR-10 Plus (Konica, Minolta, Japan). Scanning electron microscopy (SEM) images were recorded using a JSM-6700F microscope (JEOL, Musashino, Akishima, Tokyo, Japan), and confocal laser scanning microscopy images were recorded using an ECLIPSE Ti2-E confocal microscope (Nikon, Tokyo, Japan). The stress–strain curve was recorded using a texture analyzer (SurTA 1A; Chemilab Co., Seoul, South Korea).

### 2.2. Preparation of DPC Nanoparticles, S-DPC, and Dye-Incorporated DPC Shampoo (S-DPC (dye))

To synthesize the DPC, the weight ratio of HDP, GA, and PVA was optimized as shown in Table S1. Based on the optimum ratio, 70 mg of HDP, 3.5 mg of GA, and 14 mg of PVA were added into 2 mL of distilled water and stirred for 1 h at room temperature. The crude solution was stored at room temperature until two separate solutions were formed. The bottom layer was separated and then added to 30 g of shampoo, blended for 30 min at 25 °C, and marked as S-DPC. The dye-specific DPC shampoo was prepared by adding dyes that were categorized as dyes/colorants and fluorescent dyes during DPC preparation, followed by the addition of shampoo. S-DPC(red) was prepared from a mixture of DPC-shampoos, AR (30 mg) as the dye, and Rho-B (30 mg) as the fluorescent dye. S-DPC(blue) was prepared by mixing Blue 1 dye (30 mg) and TBTB fluorescent dye (30 mg) into S-DPC. S-DPC(black) was synthesized by adding NBB (30 mg), CC (15 mg), and NBB (15 mg) to S-DPC(brown). For the control trials, we prepared shampoos with no additives (S(Control)) and shampoos infused with dyes (S-red, S-blue, S-black, and S-brown) but without DPC.

### 2.3. Application of S-DPC (dye) to the Hair Samples

The dye-integrated S-DPC performance and procedure for the application of the shampoo were investigated using a hair sample with the following methods: The hair sample was pretreated by washing it in running water for 10 min and was used directly thereafter. S-DPC was evenly coated on all sections of the hair, and the process was repeated 30 times. The samples were then placed on a plastic tray for 30 min at room temperature. Subsequently, the hair samples were thoroughly rinsed under running water, placed at room temperature for 1 h, and then dried. The dyed hair was examined using a colorimeter.

### 2.4. Investigation of Anti-Electrostatic and Mechanical Properties of the S-DPC (dye)-Coated Hair Samples

The anti-electrostatic performance of the hair samples was analyzed using an air-filled rubber balloon that was stroked thrice with a cotton fabric [22]. Thereafter, the balloon was positioned near the dyed hair, and electrostatic phenomena were captured using a camera. The strain–stress curve of the coated hair samples was obtained by measuring one hair strand and placing it on the stationary grip and dynamic grip of the texture analyzer at a distance of 13.5 cm, a strain rate of 10 mm/s, and room temperature. The measurement was performed thrice for each sample (*n* = 3).

### 2.5. Determination of ROS-Scavenging Performance of S-DPC

ROS scavenging was investigated in both the dye-integrated shampoo and dye-incorporated S-DPC using DPPH, hydroxyl radical-scavenging, and superoxide radical-scavenging assays [23]. For the DPPH assay, a 0.1 mM DPPH solution (3 mL) was added to multicolor S-DPC (3 mL) and incubated at 37 °C for 30 min. DPPH inhibition was calculated using a UV-Vis spectrophotometer at a wavelength of 517 nm. For the •OH-scavenging assay, a sample (0.5 mL) was added to a mixture of salicylic acid (6 mM, 1 mL), PBS (pH 7.4, 1.5 mL), FeSO_4_ (6 mM, 1 mL), and H_2_O_2_ (0.01%, 0.5 mL). The combined solution was then incubated for 30 min at 37 °C before the absorbance was measured at 510 nm using a UV-Vis spectrophotometer. The O_2_•^−^-scavenging assay was measured by mixing the sample (0.5 mL) with 3 mL of tris-HCl buffer (pH 8.2), and 0.05 M pyrogallol solution (0.8 mL) for 5 min at 25 ℃. Subsequently, 1 mL of 8 M HCl was added, and the absorbance was measured at 325 nm using a UV-Vis spectrophotometer. The ROS-scavenging performance (DPPH, •OH, and O_2_•^−^ assay) was calculated using the following equation:

ROS scavenging performance (%) = ((A_std_ − A_sam_)/A_std_) × 100 %

where A_std_ = absorbance of standard solution and A_sam_ = absorbance of sample.

### 2.6. Cell Culture and In Vitro ROS Measurement

Human skin fibroblast Detroit 551 cells were purchased from the Korea Cell Line Bank (Seoul, Republic of Korea) and cultured in high-glucose Dulbecco’s Modified Eagle Medium with 10% fetal bovine serum, penicillin (100 units/mL), and streptomycin (100 µg/mL). Subsequently, the cells were incubated with HDP or DPC, which was initially dissolved in PBS, for a 24 h incubation period. Intracellular ROS generation was assessed using an ROS detection kit (#K936-100; Biovision, Mountain View, CA, USA) according to the manufacturer’s instructions.

### 2.7. RNA Isolation and Quantitative Real-Time Reverse-Transcription Polymerase Chain Reaction (qRT-PCR)

RNA was isolated using RNAiso Plus (#9109; Takara) according to the manufacturer’s instructions and subjected to reverse transcription using 5X All-In-One RT Master Mix (#G492; ABM). qRT-PCR was performed using AMPIGENE qPCR Green Mix Hi-ROX (Enzo, ENZ-NUC104) on a StepOne Plus real-time PCR machine (Applied Biosystems, Waltham, MA, USA). To determine the relative expression levels of the target genes, normalization to 18 S rRNA expression was performed. The following primer sequences were used:

- *CAT*: Forward: 5′-CTGGAGCACAGCATCCAATA-3′, Reverse: 5′-TCATTCAGCACGTTCACATAGA-3′;
- *NRF1*: Forward: 5′-GCCACAGCCACACATAGTATAG-3′, Reverse: 5′-CGTACCAACCTGGATAAGTGAG-3′;
- *NQO1*: Forward: 5′-GGGATGAGACACCACTGTATTT-3′, Reverse: 5′-TCTCCTCATCCTGTACCTCTTT-3′;
- *SOD1*: Forward: 5′-CTGGAACCTCACATCAAC-3′, Reverse: 5′-CTCCTGGTACTTCTCCTC-3′;
- *RN18S*: Forward: 5′-CCAGTAAGTGCGGGTCATAAG-3′, Reverse: 5′-GGCCTCACTCTA ACCATCCAA-3′.

### 2.8. Annexin V Assay

Apoptosis was assessed using a Muse cell analyzer (Merck Millipore, Billerica, MA, USA). Briefly, the cells were collected, centrifuged at 300× *g* for 5 min, and resuspended in 100 μL of Annexin V and dead cell detection reagent (#MCH100105; Merck Millipore, Billerica, MA, USA) in PBS containing 1% bovine serum albumin. This suspension was incubated at room temperature for 20 min. The analyses of the cell populations, including live, early apoptotic, late apoptotic, and dead cells, were conducted in accordance with the Millipore guidelines.

## 3. Results and Discussion

### 3.1. Design of Biocompatible and ROS-Scavenging S-DPC

**Scheme 1:**
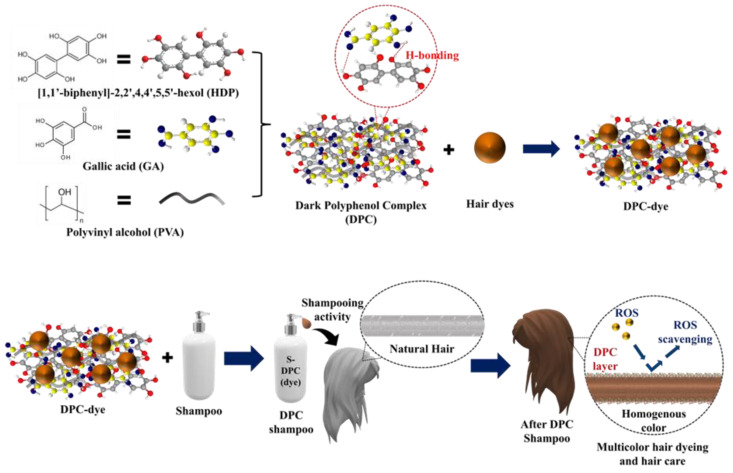
Schematic illustration of dark polyphenol complex-based dyeing shampoo (DPC shampoo) synthesis and application for homogenous hair coloring with hair-care functions such as reactive oxygen species (ROS) scavenging.

In the present study, a shampoo (S-DPC) was developed using the DPC for multicolor hair dyeing and enhancement of ROS-scavenging activity. The DPC was prepared from a mixture of PVA, GA, and HDP, and the resulting DPC played a crucial role in uniform color coating and protection of the hair from ROS (Scheme 1). HDP is important for coating stability in this system; hence, the chemical stability of HDP should be evaluated. ^1^H-NMR and high-performance liquid chromatography (HPLC) analyses were conducted to assess the chemical stability of HDP under various treatment conditions. Based on ^1^H-NMR spectra, there were no chemical shifts or changes in the area for each HDP proton peak, indicating a stable HDP structure even under differing treatment conditions (Figure S1 and Table S2). This chemical stability was further confirmed by the HPLC analysis, where there was no shift in the retention time for HDP after treatments at room temperature and under O_2_ and N_2_ atmospheres, as well as with HCl 0.1 N (Figure S2). The DPC was combined with different hair dye colors, namely, AR and Rho-B (red hair dye + red fluorescent dye; DPC(red)), Blue 1 and TBTB (blue hair dye + blue fluorescent dye; DPC(blue)), NBB (black color; DPC(black)), and CC and NBB (brown color, DPC(brown)), to demonstrate the versatility of DPC-dye for use in uniform multicolor hair dyeing. Uniform and stable dye coating on the hair was achieved owing to the presence of numerous polyphenol structures, which facilitate abundant hydrogen-bonding interactions between the DPC and the hair [24]. These hydrogen bonds contribute to improvements in the mechanical properties of the hair shafts. Furthermore, the DPC, with its polyphenol structure, results in π-π stacking, enhancing the electroconductivity and hydrophilicity of the hair shaft, which in turn helps mitigate the static charges on the hair, producing an antistatic effect [25,26,27]. Moreover, the polyphenol complex structure within the DPC acts as an antioxidant, which scavenges the ROS that are generated in the hair and thereby helps to preserve the health of the hair follicles and the scalp. The introduction of DPC-dye into a shampoo yielded a multifunctional hair-dyeing shampoo that not only ensures effective hair dyeing but also offers hair-care features that are not typically found in conventional hair dyes or hair-coloring shampoos.

**Figure 1:**
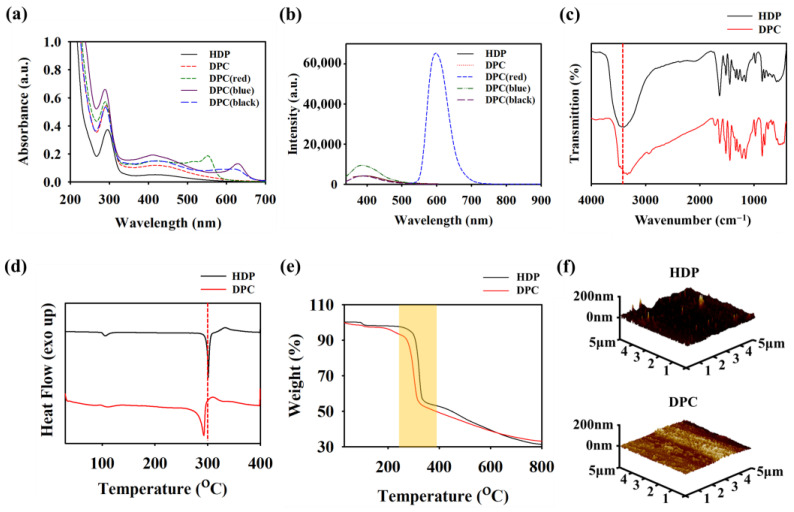
(**a**) UV-visible spectra and (**b**) photoluminescence (PL) spectra of [1,1′-biphenyl]-2,2′,4,4′,5,5′-hexol (HDP), dark polyphenol complex (DPC), DPC(red), DPC(blue), and DPC(black). (**c**) Fourier Transform infrared (FTIR) spectra, (**d**) differential scanning calorimetry (DSC) graph, (**e**) thermal gravimetric analysis (TGA) graph, and (**f**) atomic force microscopy (AFM) images of HDP and DPC.

A mixture of PVA, GA, and HDP was used to prepare the DPC colloidal nanoparticles that acted as the primary component in the multicolor hair-dyeing shampoo. HDP inherently possesses high hydrophobicity, rendering it challenging to create uniform coatings due to its propensity for aggregation and insolubility in water. The introduction of PVA and GA into the HDP suspension minimizes aggregation by reducing its hydrophobic nature. This results in a more stable and water-soluble mixture, known as DPC in water, which is important for homogenous coating and can be uniformly applied to the hair. The particle sizes of HDP and the DPC were measured using dynamic light-scattering (DLS) spectroscopy (Figure S3). The average nanoparticle diameters for HDP and the DPC were 574.0 and 369.5 nm, respectively. The larger particle size that was observed in HDP compared with that observed in the DPC indicates a greater likelihood of aggregation in HDP, which was attributed to its higher hydrophobicity. In contrast, the presence of PVA and GA in the DPC lowers its hydrophobicity, thereby reducing aggregation. The synthesized DPC was then mixed with various hair-coloring dyes (red, blue, and black), and UV-Vis spectroscopy was conducted to observe the optical and structural properties of the DPC-dyes (Figure 1a). The narrow and broad absorbance peaks at 290 and 410 nm, respectively, in DPC(red), DPC(blue), and DPC(black) were attributed to the presence of π-π* in the polyphenols of the DPC, while the peak at 570–630 nm indicated the absorption values of the hair dyes [28,29,30]. To further confirm the even distribution of the hair dye, we introduced fluorescence dyes to each DPC-dye variant, except for DPC(black), which allowed us to visualize the fluorescence on the hair after applying the DPC-dye. DPC(red) was incorporated into Rho-B, whereas DPC(blue) was modified with TBTB. The photoluminescence spectra of the DPC-dyes after incorporation of the fluorescent dyes are shown in Figure 1b. The fluorescence emission peaks of DPC(red) and DPC(blue) were detected at 597 and 391 nm, respectively. In contrast, HDP, DPC, and DPC(black) did not exhibit fluorescence, indicating that the generated fluorescence was produced by the incorporated dyes (Rho-B and TBTB) and enabling further fluorescence observation after applying the DPC-dye shampoo to the hair using, e.g., confocal microscopy.

Hydrogen bonding is essential for the uniform coating of hair dyes, and it enhances the mechanical properties of the hair. Therefore, the structural properties of the DPC were determined using FTIR spectroscopy (Figure 1c). Both HDP and the DPC possess a broad O–H stretching peak originating from polyphenols that ranges from 3000 to 3650 cm^−1^ [31]. Notably, a shift in the O–H peak was observed between HDP and the DPC, which is indicative of extensive hydrogen bonding in the DPC due to the incorporation of PVA and GA [32]. The effect of additional PVA and GA in DPC formation was also observed with DSC and TGA (heating rate: 10 °C/min; N_2_ atmosphere). Figure 1d shows the DSC graph, which reveals that the melting temperature of HDP is 300 °C, which shifted to 289 °C after being incorporated into the DPC [33]. Moreover, the TGA graph demonstrates a shift in degradation temperature from HDP (285–345 °C) to the DPC (240–315 °C) (Figure 1e). These data confirm the impact of the addition of PVA and GA during DPC synthesis. Furthermore, the presence of PVA and GA in the DPC played a major role in the increase in hydrophilicity. The AFM images show the surface roughness of HDP and the DPC, which exhibits a smoother surface than the highly hydrophobic HDP (Figure 1f). This improved hydrophilicity enabled the effective application of the DPC as a uniform hair coating.

### 3.2. Performance of Multicolor S-DPC in Hair Coloring, Strengthening, and Protection

**Figure 2:**
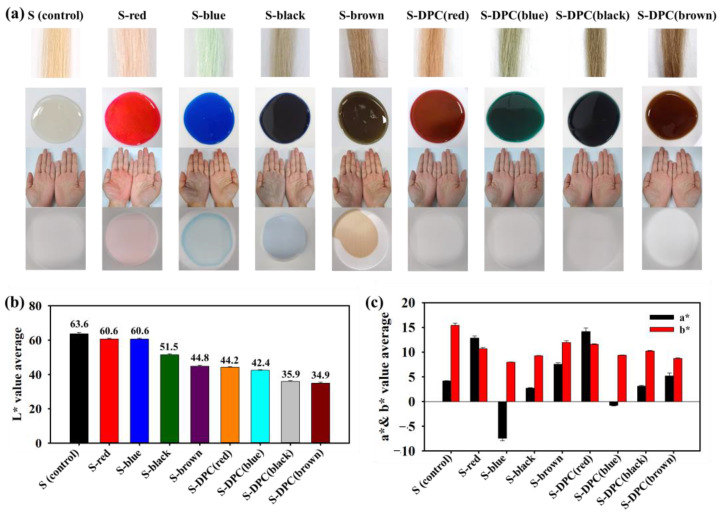
(**a**) Photographs of actual color of formulated shampoo, shampoo-treated hair, and residual color on hands and dish. (**b**) L* value and (**c**) a* and b* value measurements of shampoo only (S control), shampoo with hair dye (S-dye group: S-red, S-blue, S-black, S-brown) and dark polyphenol complex (DPC) shampoo (S-DPC(red), S-DPC(blue), S-DPC(black), and S-DPC(brown)).

After incorporating the DPC-dye into the shampoo base, S-DPC was applied to the hair model to assess homogenous coloring, strength improvement, ROS protection of the hair, and improvements in the hair’s mechanical properties. The following experimental groups were established: shampoo only (S; control), shampoo with hair dye (S-dye group: S-red, S-blue, S-black, and S-brown), and S-DPC (S-DPC(red), S-DPC(blue), S-DPC(black), and S-DPC(brown)). The colors of each shampoo group and the resulting hair color after application are shown in Figure 2a. The base shampoo/control remained colorless, whereas the addition of hair dyes (S-dye) and DPC-dyes (S-DPC) produced intense colors. After using hair-dyeing shampoos (S-dye groups), residual color frequently adhered to the hand or adhered to surfaces, such as dishes, upon contact. In contrast, compared with the S-dye groups, the S-DPC groups did not leave any residual color on the hand after shampooing or on the dish surface. This finding suggests that the presence of polyphenols in S-DPC ensures stable adherence to the hair shaft. The color characteristics can be observed using the L* (lightness), a* (red/green shade), and b* (yellow/blue shade) values of the hair after shampoo application [34]. As shown in Figure 2b, the S-DPC group (L* = 34.9–44.5) tended to be darker than the S-dye group (44.8–60.6), as indicated by the lower L* values for the S-DPC group. The a* value measurements further showed that most shampoos had a hue (positive a* value), except for S-blue and S-DPC(blue), which leaned towards greenish tones (negative a* value). As for b* values, all shampoos showed yellowish tones that are characteristic of positive values. Notably, the S-DPC group (b* = 8.7–11.9) exhibited a more pronounced yellow shade than the S-dye group (b* = 7.9–12.8) (Figure 2c and Table S3).

**Figure 3:**
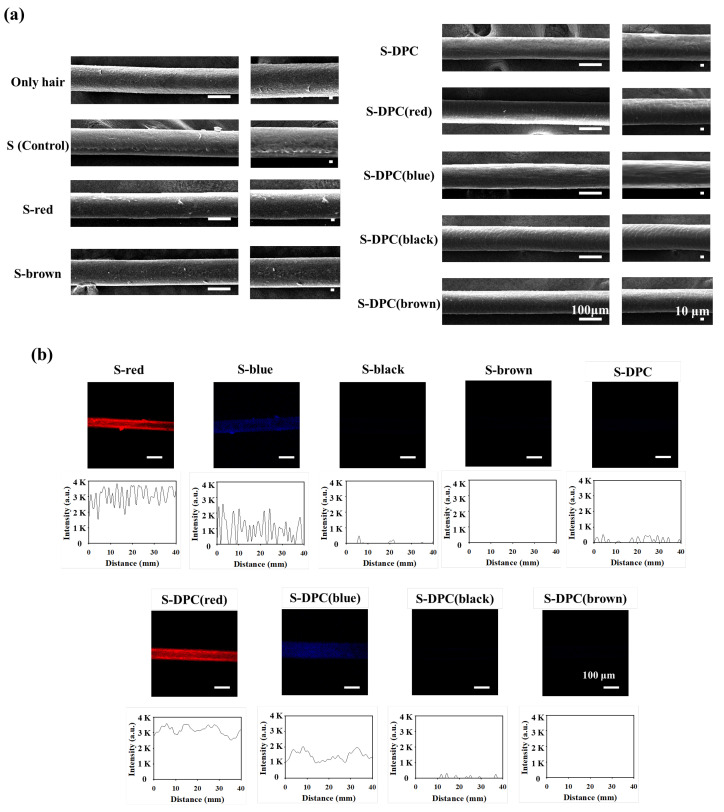
(**a**) Scanning electron microscopy (SEM) images and (**b**) confocal images of hair treated with shampoo only (S control), S-dyes, and dark polyphenol complex (DPC)-integrated shampoo (S-DPC).

S-DPC can uniformly coat and color human hair owing to the adhesive properties of its constituent polyphenols. SEM images demonstrated differences in hair dye coating results after the shampooing process in the S (control), S-dye, and S-DPC groups (Figure 3a). Initially, the hair exhibited a rough cuticle surface. However, after the application of S-DPC, the hair surface became smoother and less aggregated, as the DPC-dyes were uniformly coated on the hair shaft [35]. Conversely, the SEM images of the S (control) and S-dye groups reveal a partially rough surface and uneven dye coating. The difference in the homogeneity of hair coating was also observed using confocal microscopy, as the fluorescent dyes (Rho-B: red; TBTB: blue) were incorporated into the S-dye and S-DPC groups prior to this measurement. As shown in Figure 3b, S-red and S-blue showed uneven fluorescence distribution on the hair due to non-uniform coating. In contrast, S-DPC(red) and S-DPC(blue) exhibited well-distributed red and blue fluorescence, confirming the uniform coating of DPC-dyes on the hair [36]. Furthermore, the cross-section confocal images of S-DPC(dye)-treated hair reveal that the color was retained on the surface of the hair rather than penetrating to the center of the hair fiber. This result confirms that the principle of our hair coloring method is based on surface coating as opposed to dye penetration owing to the coating stability of polyphenols (Figure S4). The color coating stability of S-DPC was assessed after washing the hair 10 times. The confocal images of S-DPC(red) and S-DPC(blue)-treated hair showed retained fluorescence intensity even after exposure to multiple washing processes, indicating the stability of the S-DPC hair color (Figure S5).

**Figure 4:**
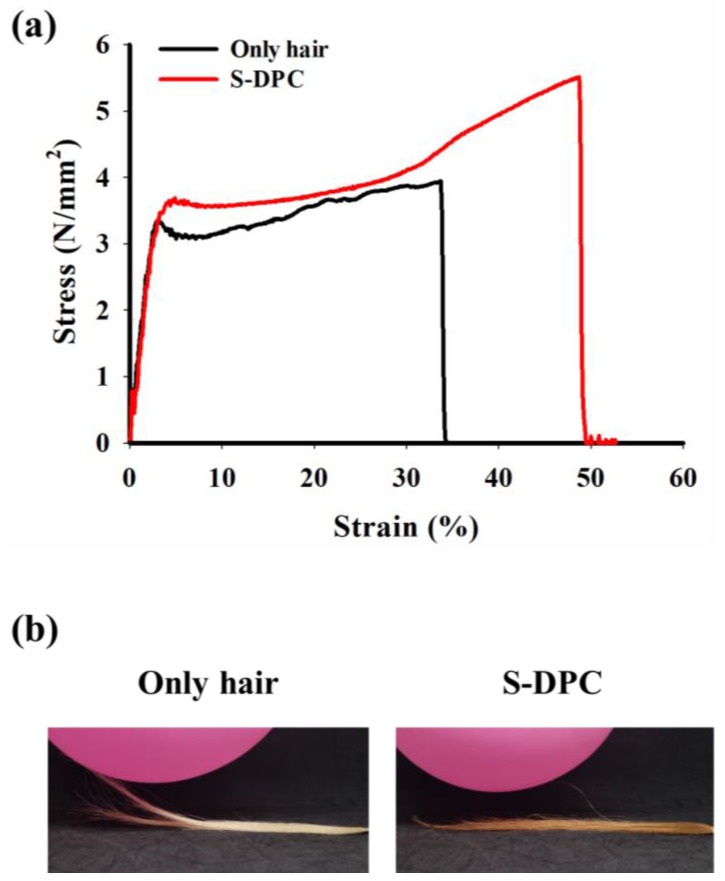
(**a**) Tensile strength and (**b**) antistatic effect of untreated hair and dark polyphenol complex (DPC)-integrated shampoo (S-DPC)-treated hair.

Owing to its high polyphenol content, S-DPC can improve the mechanical strength of the hair shafts. Tensile strength analysis was conducted using a universal testing machine to measure the enhancement in hair strength with and without S-DPC treatment (Figure 4a). The tensile strain and stress of S-DPC-treated hair were increased significantly (48.76% and 5.54 N/mm^2^, respectively) compared with those of untreated hair (33.78% and 3.96 N/mm^2^, respectively). This improvement in mechanical properties after S-DPC treatment was due to the abundance of hydrogen bonding as well as π-π stacking in the DPC. Furthermore, the inclusion of the DPC in the shampoo imparted an antistatic effect on the treated hair. Static hair, often resulting from the triboelectric effect, is a common issue, particularly in cold and dry environments [37]. The occurrence of electrostatic charges on the hair triggers entanglement and repulsion between hair molecules, damaging the hair and causing inconvenience. Therefore, the antistatic property of S-DPC plays an important role in addressing this issue by dissipating electrostatic charges on the hair. To observe the antistatic effects, we compared S-DPC-treated hair with untreated hair that was placed near a rubbed balloon. As shown in Figure 4b, the S-DPC-treated hair containing the DPC from the shampoo displayed a notable antistatic effect. This was evident, as the hair did not cling to the rubber balloon because of static induction. In contrast, the untreated hair adhered to the rubber balloon, indicating a lack of antistatic properties. This result suggests that the dissipation of electrostatic charges on the S-DPC-treated hair was attributable to π-π stacking, which can increase conductivity, thereby facilitating effective charge dissipation on the hair. The hair may have also been affected by the increase in hydrophilicity after its coating with S-DPC, which reduced surface resistivity on the hair [38,39].

**Figure 5:**
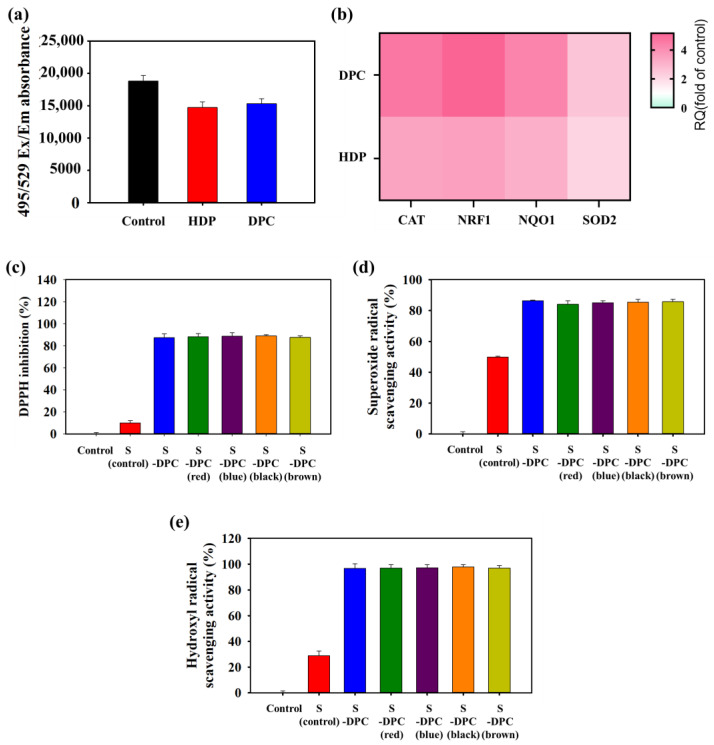
(**a**) Intracellular reactive oxygen species (ROS) levels and (**b**) the expression levels of genes involved in the antioxidant pathway, including catalase (*CAT*), nuclear factor erythroid-derived 2-related factor 1 (*NRF1*), NAD(P)H:quinone acceptor oxidoreductase 1 (*NQO1*), and superoxide dismutase 2 (*SOD2*), within Detroit 551 cells after treatment with 0.4 μg/mL HDP or 0.8 μg/mL DPC for 24 h. (**c**) DPPH, (**d**) superoxide (O_2_^−^) radical-scavenging, and (**e**) hydroxide (OH) radical-scavenging assays after treatment with S control and DPC-integrated shampoo (S-DPC).

**Figure 6:**
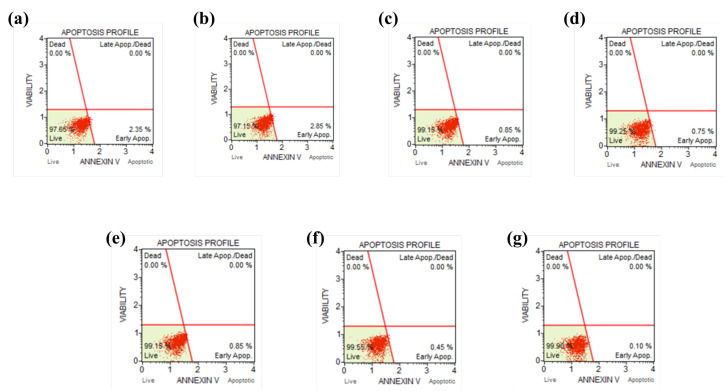
Apoptotic cell death assessed with flow cytometry with the Annexin V staining of Detroit 551 cells (**a**) without treatment and treated with (**b**) 0.2 μg/mL, (**c**) 0.4 μg/mL, and (**d**) 0.8 μg/mL HDP, as well as (**e**) 0.4 μg/mL, (**f**) 0.8 μg/mL, and (**g**) 1.6 μg/mL DPC, respectively, for 24 h.

The antioxidant effects of the DPC were examined by assessing ROS generation in Detroit 551 cells that were treated with the DPC (or HDP) in the presence of an ROS inducer. The results show a substantial reduction in cellular ROS levels within Detroit 551 cells after a 24 h treatment with 0.4 μg/mL HDP or 0.8 μg/mL DPC (Figure 5a). Furthermore, exposure of the cells to HDP or the DPC increased the expression levels of the genes involved in antioxidant pathways, including catalase (*CAT*), nuclear factor erythroid-derived 2-related factor 1 (*NRF1*), NAD(P)H:quinone acceptor oxidoreductase 1 (*NQO1*), and superoxide dismutase 2 (*SOD2*) (Figure 5b). The ROS-scavenging efficiency of the DPC nanoparticles was evaluated against DPPH, O_2_•^−^, and •OH. The results reveal that the DPC nanoparticles demonstrated remarkable ROS-scavenging efficiency, approximately 62.4–64.5% for DPPH (Figure S6a), 66.5–67.8% for O_2_•^−^ (Figure S6b), and 89.8–90.8% for •OH (Figure S6c). Furthermore, the ROS-scavenging activity of S-DPC was also determined with the DPPH assay (Figure 5c and Figure S7) [40]. S-DPC possessed high ROS-scavenging efficiency, as reflected by high DPPH inhibition (~90%), compared with that without DPC (S control/ shampoo only), which showed negligible DPPH inhibition (~10%). S-DPC also effectively scavenged O_2_^-^ and OH radicals. Figure 5d shows a significant difference in O_2_•^−^-scavenging efficiency between the S-DPC (84–86%) and S control groups (49%), whereas Figure 5e shows the superiority of S-DPC (96.6–97.7%) in scavenging •OH compared with the S control (28.9%). Annexin V flow cytometry was performed to investigate the potential effects of apoptosis on HDP- and DPC-induced cell toxicity. The results reveal that treatment with HDP or the DPC in a dose-dependent manner did not alter the percentage of total apoptotic cells compared with that of the control cells, suggesting no cytotoxic activity toward human skin fibroblasts (Figure 6). These findings underscore the role of the DPC in shampoos as an effective ROS scavenger with high biocompatibility.

## 4. Conclusions

We successfully developed S-DPC, a shampoo with integrated DPC, for uniform multicolor hair dyeing with the added benefits of protection against ROS and enhanced hair strength. The mixture of HDP, GA, and PVA, which forms a DPC structure, facilitates extensive hydrogen bonding that promotes a stable (no residual color) and uniform coating of the dye when combined with various hair colorants and shampoo formulations. S-DPC also improved the mechanical properties of the hair, as indicated by higher tensile strain and stress (48.76% and 5.54 N/mm^2^) compared with untreated hair (33.78% and 3.96 N/mm^2^). Moreover, the presence of the DPC in the shampoo conferred an effective antistatic effect that effectively dissipated the electrostatic charges on the hair by increasing its conductivity. In addition to its mechanical benefits, S-DPC provided remarkable ROS protection, showing high scavenging efficiency in DPPH (90%), O_2_•^−^ (84–86%), and •OH assays (96.6–97.7%). Importantly, cytotoxicity tests confirmed the biocompatibility of the shampoo, with no observed harmful effects on human skin fibroblasts. Therefore, the introduction of the DPC was crucial to the development of an innovative multicolor hair-dyeing shampoo with hair-care functions, which offers a major advantage over conventional hair colorants and hair-coloring shampoos.

## Data Availability

Data available on request from the authors.

## Supplementary Materials

The following supporting information can be downloaded at: https://www.mdpi.com/article/10.3390/biomimetics8060469/s1, Figure S1: ^1^H-NMR spectra of HDP treated under the conditions of RT, O_2_, N_2_, and HCl 0.1 N, Figure S2: HPLC graph of HDP treated under the conditions of RT, O_2_, N_2_, and HCl 0.1 N, Figure S3: DLS spectra of HDP and DPC, Figure S4: Confocal image of cross-section hair and S-DPC (dye)-treated hair, Figure S5: Confocal image of S-DPC (dye)-treated hair after being subjected to the frequent-washing process (10 times), Figure S6: (a) DPPH, (b) superoxide radical (O_2_•^−^)-scavenging, and (c) hydroxyl radical (•OH)-scavenging assays of HDP and DPC nanoparticles, Figure S7: Visual of DPPH assay using DPC nanoparticle and DPC shampoo, Table S1: Optimization of DPC synthesis based on weight ratio of HDP, GA, and PVA, Table S2: Proton (1H) peak area of HDP treated with various parameters, Table S3: *a** and *b** values of shampoo only (S control), shampoo with hair dye (S-dye group: S-red, S-blue, S-black, S-brown), and dark polyphenol complex (DPC) shampoo (S-DPC(red), S-DPC(blue), S-DPC(black), S-DPC(brown)).
