# Supplementary material for: Multicolor Hair Dyeing with Biocompatible Dark Polyphenol Complex-Integrated Shampoo with Reactive Oxygen Species Scavenging Activity
Source: Biomimetics (Basel). 2023 Oct 1;8(6):469. doi: 10.3390/biomimetics8060469 (PMC10604431; doi:10.3390/biomimetics8060469)
Supplement: Supplementary file 1 [file biomimetics-08-00469-s001.zip › biomimetics-2628034-supplementary.pdf]

SUPPORTING INFORMATION

Multicolor Hair Dyeing with Biocompatible Dark Polyphenol Complex-Integrated Shampoo with Reactive Oxygen Species Scavenging Activity

Tae Min Kim [1,†], Hyun Jeong Won [1,†], Jun-Ho Yang [2,†], Hayeon Jo [1], A Hyeon Kim [3], Dohyun Nam [2], Seul Gi Kim [1], Eun-Jung Jin [2,*], Heung Jin Bae [4,*] and Sung Young Park [1,3,*]

[1] Department of Chemical and Biological Engineering, Korea National University of Transportation,
Chungju 27469, Republic of Korea; ktm1120@a.ut.ac.kr (T.M.K.); dnjsswh@ut.ac.kr (H.J.W.); yeon81037@ut.ac.kr (H.J.); k.seulgi9797@gmail.com (S.G.K.)

[2] Department of Biological Sciences, College of Health Sciences, Wonkwang University, Iksan 54538, Republic of Korea; skyjun96@gmail.com (J.-H.Y.); dhnam0120@gmail.com (D.N.)

[3] Department of IT and Energy Convergence (BK21 FOUR), Korea National University of Transportation, Chungju 27469, Republic of Korea; dkgusaks@naver.com

[4] MODAMODA Corporation, Ltd., Songpa-gu, Seoul 05546, Republic of Korea

* Correspondence: jineunjung@wku.ac.kr (E.-J.J.), newseowha@naver.com (H.J.B.) parkchem@ut.ac.kr (S.Y.P.)

† These authors contributed equally to this work.

1. EXPERIMENTAL SECTION

1.1. Investigation of HDP stability using ^{1}H-NMR and HPLC

Prior to the investigation, the sample was pre-treated using the following procedures. The HDP sample was dissolved in distilled water with a final concentration of 0.4 mg/mL and stirred for 7 days at 200 rpm. Each sample was treated at different treatment conditions such as room temperature (RT) without and with O_2, N_2, and 0.1 N HCl. The solution was lyophilized before analysis.

^{1}H-NMR was analyzed using Bruker Avance 400 MHz spectrometer using deuterated-dimethyl sulfoxide (DMSO-d_6) as the solvents and the concentration of 10 mg/mL. The HPLC was recorded at F&F Tech Co., Ltd. using methanol (MeOH) and 0.1% KH_2PO_4 in water for HPLC as the mobile phase. OptimapakC-18 (4.6 × 250mm) was used as the stationary phase and the analysis was performed at a measurement temperature of 35 °C, flowrate at 0.8 mL/min, and detection wavelength of 290 nm.

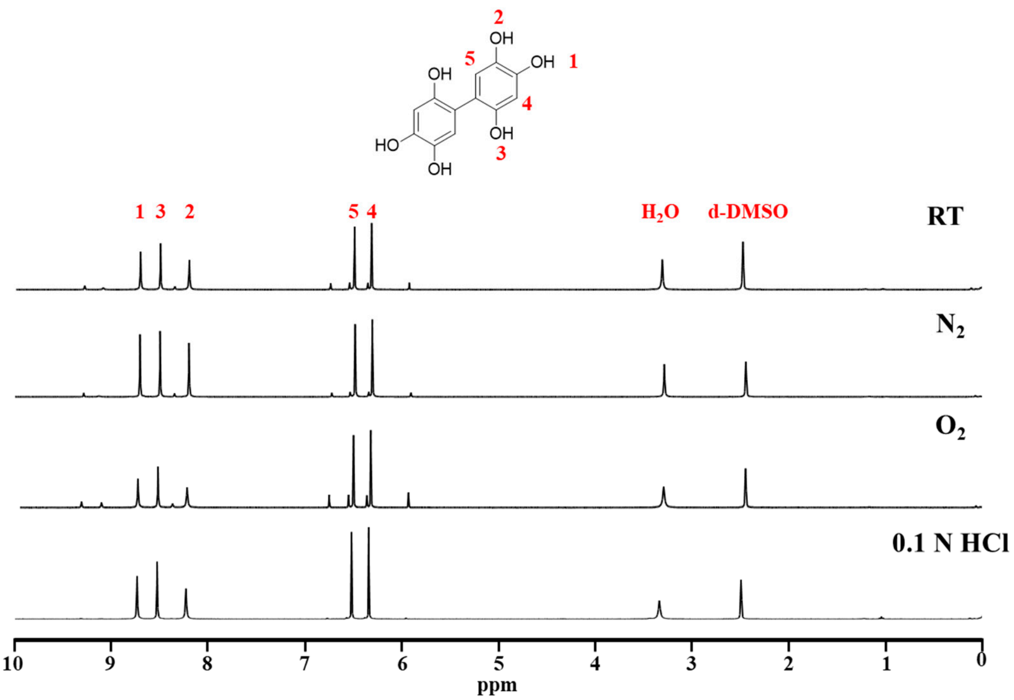

Figure S1. ^{1}H-NMR spectra of HDP treated in RT, O_2, N_2, and HCl 0.1 N.

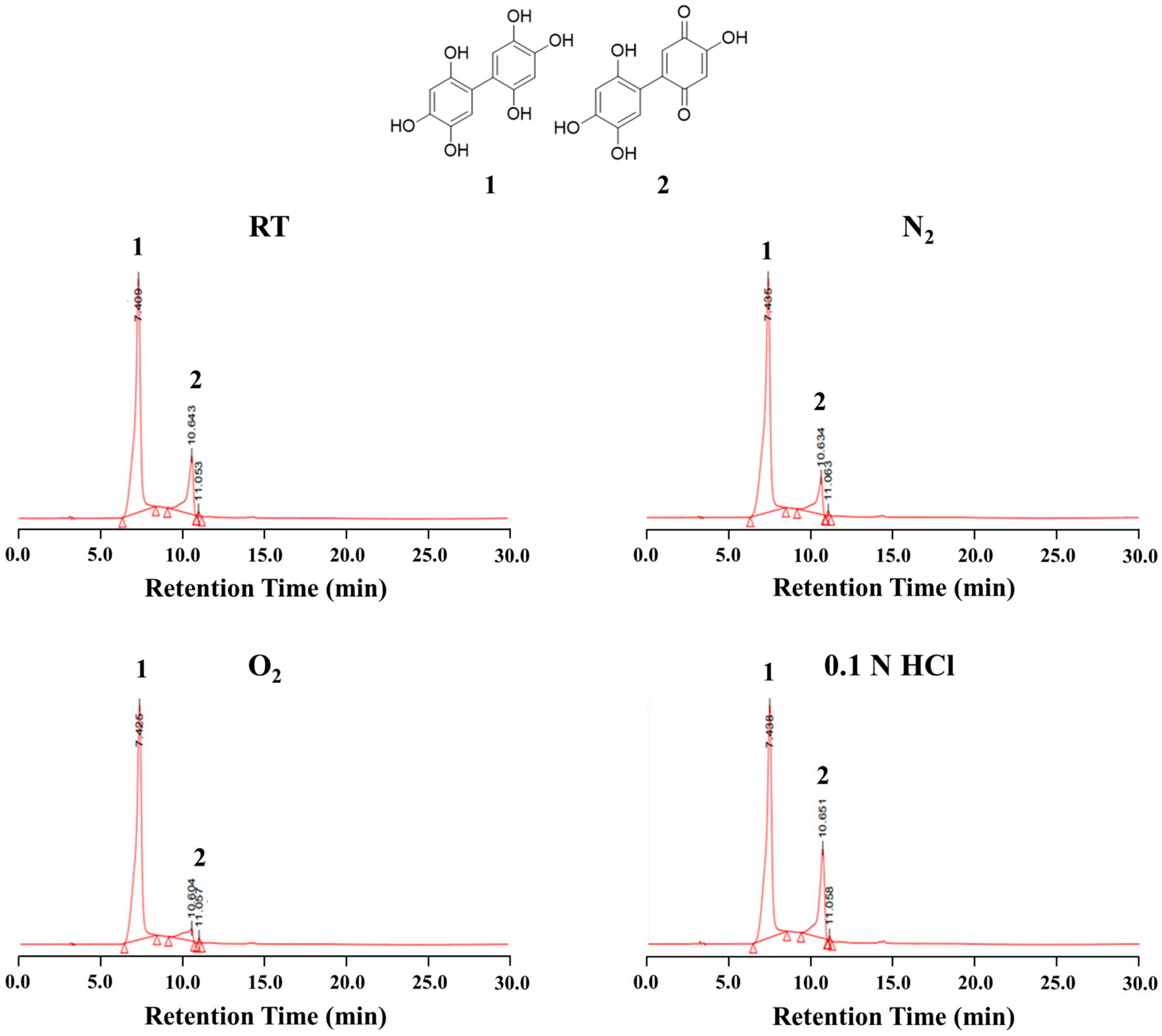

Figure S2. HPLC graph of HDP treated in RT, O_2, N_2, and HCl 0.1 N.

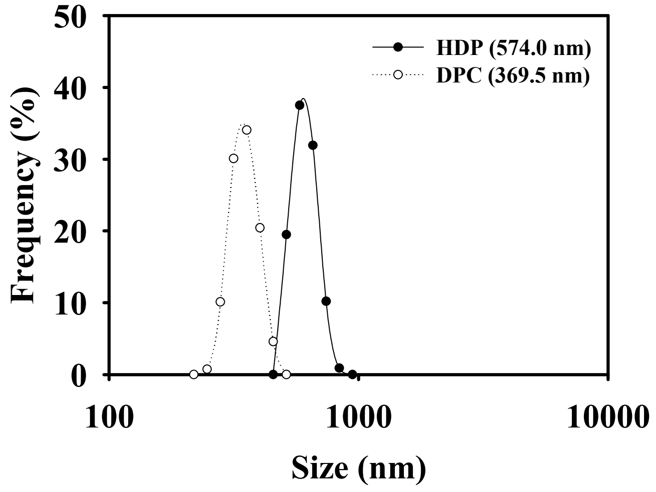

Figure S3. DLS spectra of HDP and DPC.

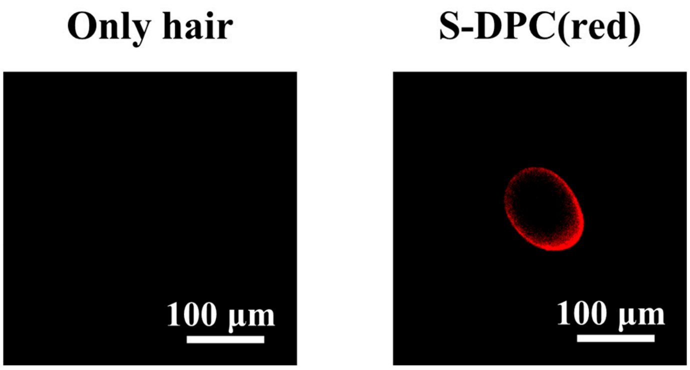

Figure S4. Confocal image of cross-section hair and S-DPC(dye)-treated hair.

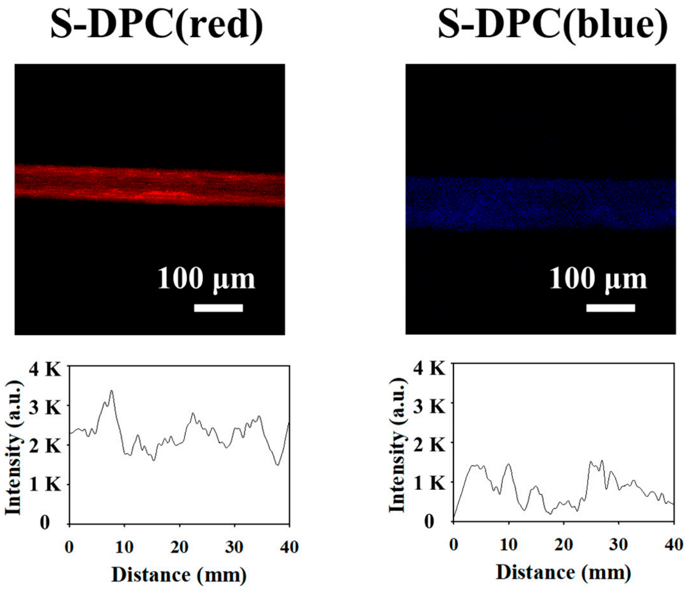

Figure S5. Confocal image of S-DPC(dye)-treated hair after subjected to the frequent washing process (10 times).

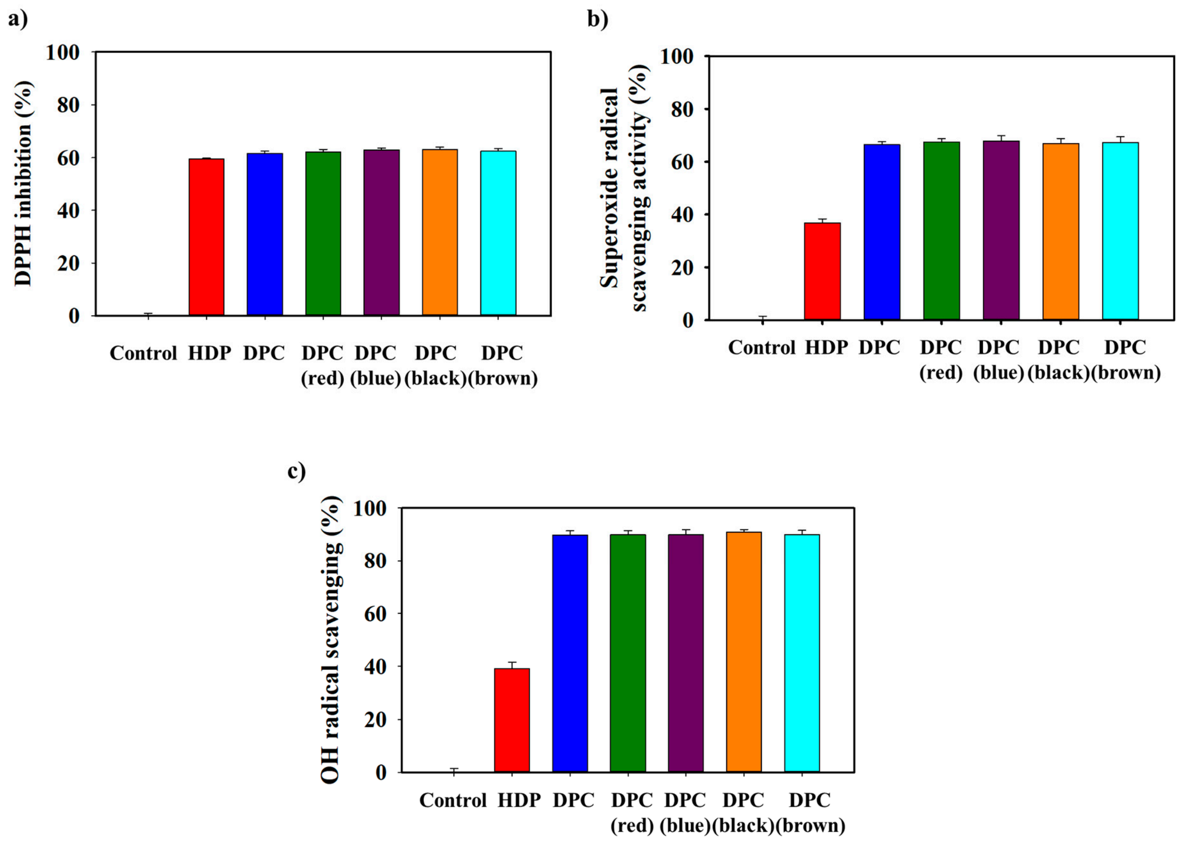

Figure S6. a) DPPH, **b)** superoxide ($O_2^{\bullet-}$) and **c)** hydroxyl radical (•OH) scavenging assays of HDP and DPC nanoparticles.

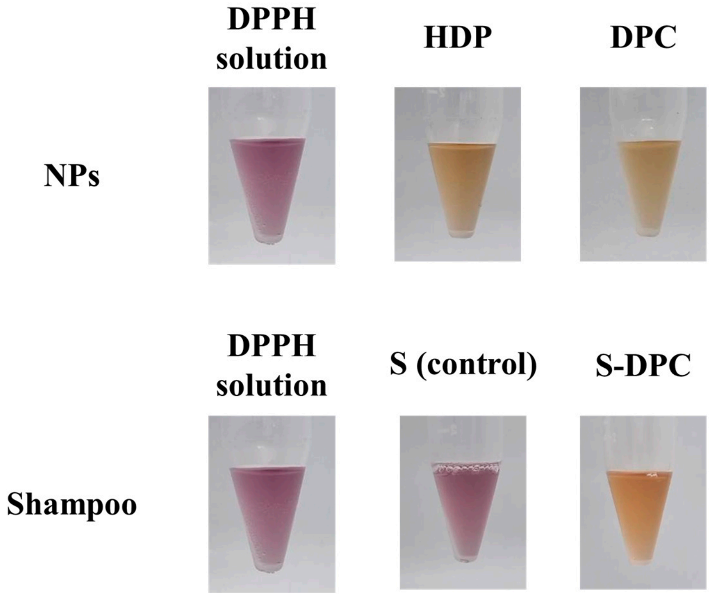

Figure S7. Visual of DPPH assay using DPC nanoparticle and DPC shampoo.

Table S1. Optimization of DPC synthesis based on weight ratio of HDP, GA and PVA.

No.	HDP (mg)	GA (mg)	PVA (mg)	DDW (mL)
1	30	3.5	14	2
2	70	3.5	14	2
3	140	3.5	14	2

Table S2. Proton (^{1}H) peak area of HDP treated with various parameters.

**H No.**	**H peak area of HDP**			
	**RT**	**N_2**	**O_2**	**0.1 N HCl**
**1**	1.0	1.0	1.0	1.0
**2**	1.0	1.0	1.0	1.0
**3**	1.0	1.0	1.0	1.0
**4**	1.1	1.1	1.2	1.0
**5**	1.1	1.1	1.2	1.0

Table S3. *a** and *b** values of shampoo only (S control), shampoo with hair dye (S-dye group: S-red, S-blue, S-black, S-brown) and dark polyphenol complex (DPC) shampoo (S-DPC(red), S-DPC(blue), S-DPC(black), S-DPC(brown)).

	a* **value average**	***b**** **value average**
**S (control)**	4.2	15.4
**S-red**	12.8	10.7
**S-blue**	-7.5	7.9
**S-black**	2.7	9.3
**S-brown**	8.4	12.8
**S-DPC(red)**	14.1	11.6
**S-DPC(blue)**	-0.8	9.4
**S-DPC(black)**	3.1	10.2
**S-DPC(brown)**	5.2	8.7
